# Anaplastic lymphoma kinase inhibitors—a review of anticancer properties, clinical efficacy, and resistance mechanisms

**DOI:** 10.3389/fphar.2023.1285374

**Published:** 2023-10-25

**Authors:** Kajetan Kiełbowski, Justyna Żychowska, Rafał Becht

**Affiliations:** Department of Clinical Oncology, Chemotherapy and Cancer Immunotherapy, Pomeranian Medical University, Szczecin, Poland

**Keywords:** anaplastic lymphoma kinase, ALK inhibitor, tyrosine kinase inhibitor, resistance mechanisms, personalized oncology

## Abstract

Fusions and mutations of anaplastic lymphoma kinase (ALK), a tyrosine kinase receptor, have been identified in several neoplastic diseases. Rearranged ALK is a driver of tumorigenesis, which activates various signaling pathway associated with proliferation and survival. To date, several agents that target and inhibit ALK have been developed. The most studied ALK-positive disease is non-small cell lung cancer, and three generations of ALK tyrosine kinase inhibitors (TKIs) have been approved for the treatment of metastatic disease. Nevertheless, the use of ALK-TKIs is associated with acquired resistance (resistance mutations, bypass signaling), which leads to disease progression and may require a substitution or introduction of other treatment agents. Understanding of the complex nature and network of resistance mutations may allow to introduce sequential and targeted therapies. In this review, we aim to summarize the efficacy and safety profile of ALK inhibitors, describe off-target anticancer effects, and discuss resistance mechanisms in the context of personalized oncology.

## 1 Introduction

**FIGURE 1:**
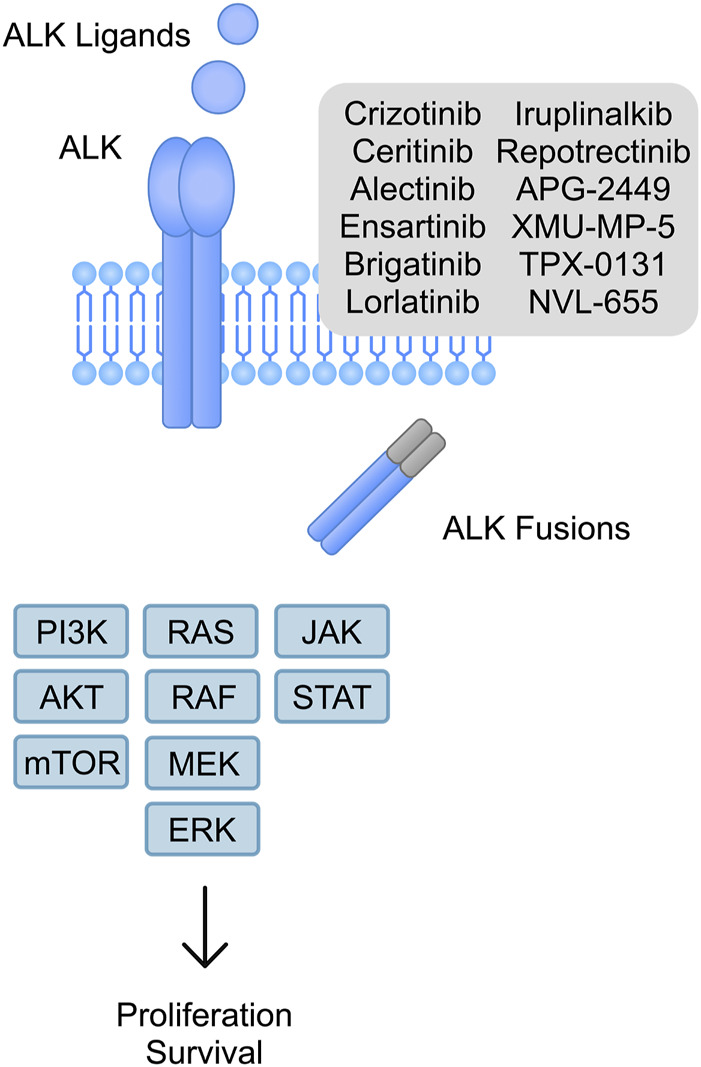
A schematic illustration of ligand dependent ALK tyrosine kinase receptor and ALK fusions, which stimulate signaling pathways associated with proliferation and survival.

Oncogenesis is a complex process associated with activation of oncogenes and inhibition of tumor suppressors. This leads to a constant cell proliferation and inhibition of apoptosis. Anaplastic lymphoma kinase (ALK) is a membrane tyrosine kinase receptor, which was identified for the first time in 1994 (Morris et al., 1994). The broad family of tyrosine kinase receptors plays a crucial role in cellular signaling and its abnormal activation due to receptor mutations is a long-known process of tumorigenesis (Lemmon and Schlessinger, 2010). In the case of ALK, it stimulates multiple signaling pathways associated with cell growth, including janus kinase-signal transducer and activator of transcription (JAK-STAT), mitogen activated protein kinases (MAPK), and phosphatidylinositol-3-kinase/protein kinase B (PI3K/AKT) pathways, among others (Figure 1). Numerous fusion partners of ALK have been identified (e.g., nucleophosmin (NPM)-ALK in anaplastic large cell lymphoma (ALCL) and echinoderm microtubule-associated protein-like 4 (EML4)-ALK in non-small cell lung cancer (NSCLC)). Furthermore, point mutations in the ALK receptor have been found in neuroblastoma and anaplastic thyroid cancer as well (Hallberg and Palmer, 2016). The aim of this review is to summarize generations of ALK tyrosine kinase inhibitors (TKI), present their efficacy and anti-cancer properties, and to discuss various mechanisms of resistance.

## 2 Anaplastic lymphoma kinase–structure and expression

The structure of ALK includes an extracellular domain (with two MAM and LDL-A domains), glycine-rich, transmembrane, and protein tyrosine kinase domains (Lee et al., 2010). Normal ALK protein is expressed at relatively low levels in most of the tissues, except for nervous system (Wu et al., 2017). While normal protein has extracellular and transmembrane domains, the majority of rearranged ALK proteins are considered to localize intracellularly (Chiarle et al., 2008; Sabir et al., 2017). Rearrangements of ALK have been broadly studied in the case of NSCLC. However, studies showed conflicting results regarding ALK expression rates, as populations with various baseline characteristics were examined. According to Shaw et al. (2009), out of 141 tumors, 19 (13%) were EML4-ALK positive. In separate studies by Zhang et al. (2016); Aguado de la Rosa et al. (2022), ALK rearrangements were observed in 5% and 5.5% of patients, respectively. Clinically, ALK rearrangement is associated with younger age, no history of smoking, advanced stage, and occurs more often in females (Fan et al., 2014; Aguado de la Rosa et al., 2022). Interestingly, these aberrations are associated with higher survival in surgically treated lung adenocarcinomas (Matsuura et al., 2023). EML4-ALK is the most-studied fusion in NSCLC. Based on the fusion breakpoints, several structurally different EML4-ALK variants have been identified. In NSCLC, variants 1 and 3a/b are the most common and presence v1 has been previously associated with improved clinical outcomes (Zhang et al., 2021). Importantly, as more advanced genetic testing methods are becoming more popular (e.g., next-generation sequencing (NGS)), multiple novel ALK-fusions have been identified. In 2020, approximately 90 ALK fusion partners in NSCLC have been described (Ou and Nagasaka, 2020). Moreover, ALK rearrangements have been identified in ALCL (translocation, 60%) (Falini et al., 1998), thyroid cancer (translocation, 2.2%) (Chou et al., 2015), and neuroblastoma (point mutation, 10.5%) (Rosswog et al., 2023), among others.

## 3 Anaplastic lymphoma kinase inhibitors

### 3.1 Anaplastic lymphoma kinase–I generation inhibitors

Crizotinib represents the first generation of ALK inhibitors, which was approved by the FDA for the treatment of NSCLC, as well as ALCL in children and young adults. It is a multitarget TKI, which inhibits ALK, mesenchymal epithelial transition growth factor (c‐MET) and ROS proto-oncogene 1 receptor kinase (ROS1) (Sahu et al., 2013). It prevents ATP from binding to ALK and its subsequent autophosphorylation (Bang, 2011). The drug showed antitumor activity in preclinical studies, as well as promising results in phase I clinical trials (Forde and Rudin, 2012). Crizotinib showed better outcomes (progression-free survival (PFS), partial response (PR)) in ALK-positive NSCLC compared to chemotherapy in phase 3 clinical trials (as a second- and first-line treatment agent). (Shaw et al., 2013; Solomon et al., 2014). In the study by Solomon et al. (2014) 13 reactions occurred more frequently in the crizotinib group than in chemotherapy. In contrast, 7 events were more common in chemotherapy cohort, while frequency of five reactions was similar between groups. Overall, the two most common adverse events (AEs) in the crizotinib group included vision disorder (71%), and diarrhea (61%). Importantly, these AEs rarely developed in grade 3–4 (1% and 2%, respectively). The most frequent grade 3–4 AEs in the study group included elevated aminotransferases (14%) and neutropenia (11%) (Solomon et al., 2014). Less is known about the efficacy of crizotinib in tumors other than NSCLC. Nevertheless, good response was presented in reports of patients with thyroid cancers (Godbert et al., 2015; de Salins et al., 2020) and inflammatory myofibroblastic tumors (IMT) (Kimbara et al., 2014; Ogata et al., 2019). According to the phase 2 study by Schöffski et al. (2021), objective response rate (ORR) was 66.7% in patients with ALK-positive IMT and 14.3% in ALK-negative tumors. Furthermore, in ALK-positive cohort, improved median PFS was observed (18 vs. 14.3 months).

**FIGURE 2:**
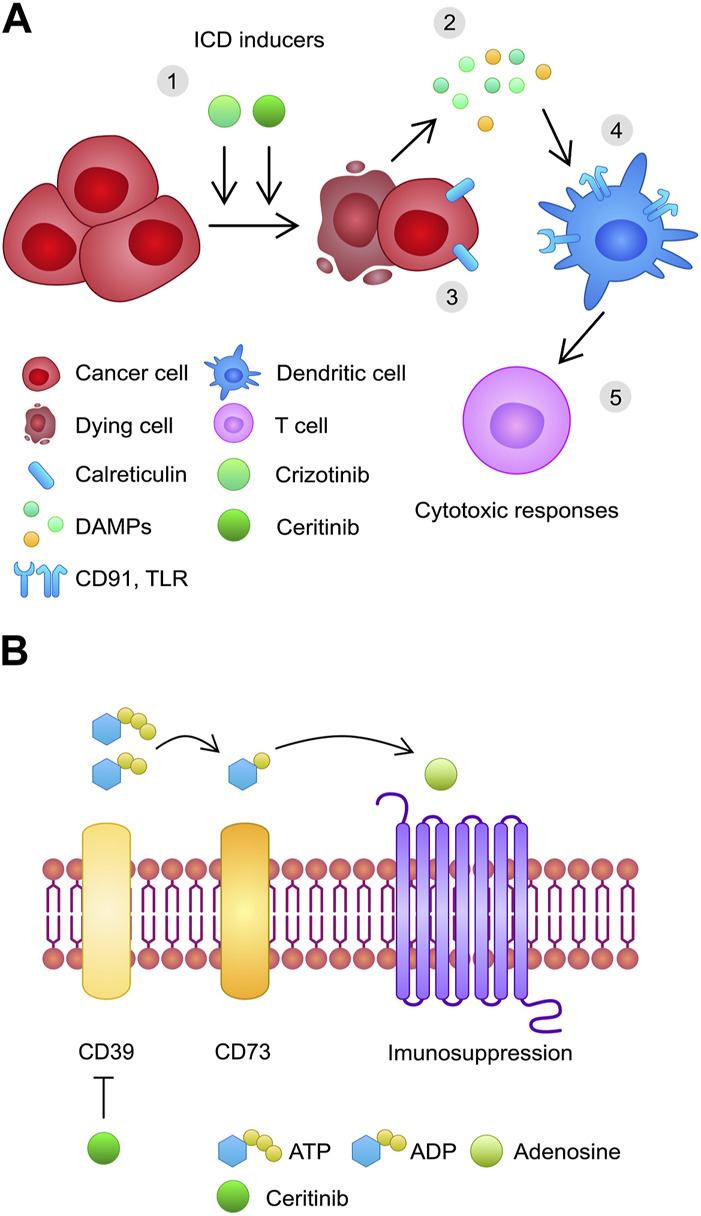
A schematic illustration of additional anti-cancer mechanisms mediated by ALK inhibitors based on research performed by Liu et al. (2019); Petrazzuolo et al. (2021); Schäkel et al. (2022). **(A)** 1) Crizotinib and ceritinib in cells induce immunogenic cell death through 2) the release of DAMPs and 3) expression of calreticulin, 4) which stimulates dendritic cells and 5) T cells, inducing cytotoxic responses. **(B)** Ceritinib inhibits ectonuclease CD39, which prevents adenosine development and immunosuppressive actions.

Interestingly, ALK inhibitors may induce a process known as immunogenic cell death (ICD). Dying tumor cells release signals engaging the immune system, such as damage-associated molecular patterns (DAMPs), including ATP or high-mobility group box-1 (HMGB1). Furthermore, expression of certain proteins, such as calreticulin, promotes the activity of dendritic cells, and subsequently T cells (Ahmed and Tait, 2020). Liu et al. (2019) showed that crizotinib added to a human NSCLC line expressing EML4-ALK, resulted in a significant increase of calreticulin exposure, together with HMGB1 and ATP release. However, high concentrations of crizotinib induced hallmarks of ICD in EML4-ALK negative NSCLC cell lines (Figure 2). Moreover, a combination of standard chemotherapeutics with crizotinib induced immunogenicity in cancer model *in vivo*. Furthermore, crizotinib combined with cisplatin induced the expression of immune checkpoints *in vivo* and *in vitro*, which resulted in improved results of combination of crizotinib and immune checkpoint inhibitors (Liu et al., 2019). Furthermore, it has been demonstrated that EML4-ALK fusion induces the expression of PD-L1 through the PI3K and MEK/ERK pathways (Ota et al., 2015). Zhou and others investigated OS and PFS of 128 NSCLC patients treated with first-line crizotinib and divided them into two cohorts based on the PD-L1. Higher expression was associated with significantly worse PFS and OS (median 6 vs. 11 months, 17 vs. 53 months, respectively) (Zhou et al., 2022). In addition, an evaluation of 1001 NSCLC patients revealed that ALK and ROS1 rearrangement was associated with higher expression of PD-L1 (Chu et al., 2022). These results suggest that combination of ALK-TKIs with ICIs might provide good efficacy. Only a few studies examined this combination, but they showed significant AEs in included patients. Lin et al. described eleven patients treated sequentially with ICI and crizotinib. Five patients (45.5%) experienced grade 3/4 elevation of ALT, compared to 34 (8.1%) patients treated with crizotinib alone. Similarly, high AST concentrations (grade 3/4) were observed in the group with sequential treatment (36.4% vs. 3.4%) (Lin et al., 2019). Significant hepatotoxicity was also reported in studies evaluating crizotinib combined with nivolumab or pembrolizumab as a first-line treatment for advanced NSCLC (Spigel et al., 2018; Patel et al., 2020). Interestingly, crizotinib has been recently associated with another anti-cancer mechanism. Burikhanov et al. showed that crizotinib increased the expression of GRP78 (csGRP78), a cancer-related receptor, which triggers survival-related signaling pathways in KRAS-, NRAS, and EGFR-mutant NSCLC cells. Furthermore, the authors showed that crizotinib promoted a secretion of prostate apoptosis response-4 (Par-4) in normal cells, which binds to csGRP78 and triggers apoptosis. In mice models with ALK-negative tumor, crizotinib showed antitumor efficacy in PAR-4 ^+/+^ animals, while it did not inhibit the growth of tumor in PAR-4 ^−/−^ mice. Therefore, crizotinib might have antitumor efficacy in both ALK-positive and negative cancers (Burikhanov et al., 2023).

Importantly, crizotinib has been investigated in combination with an anti-angiogenic agent. Angiogenesis is a significant mechanism in the process of tumorigenesis. Intratumoral blood vessels provide nutrients and oxygen supply to rapidly proliferating malignant cells. Vascular endothelial growth factor (VEGF) is a major pro-angiogenic mediator. Consequently, anti-angiogenic agents target VEGF or its receptors to inhibit the formation of new intratumoral vessels. Huang et al., described the safety and efficacy of crizotinib combined with bevacizumab, a monoclonal antibody targeting VEGF, in 14 patients with NSCLC, among whom ALK rearrangements were detected in 12 patients. The median PFS in a group of ALK-positive patients was 13.9 months, while the median OS was not reached. Therefore, this combination showed improved PFS than crizotinib monotherapy described in the PROFILE 1014 trial. The most common AEs in this combination were fatigue and rash, which occurred in 28.6% and 21.4%, respectively (Huang et al., 2021).

### 3.2 Anaplastic lymphoma kinase–II generation inhibitors

Several second-generation ALK TKIs have been developed to increase efficacy and overcome resistance to crizotinib. Ceritinib was approved by the FDA for the treatment of ALK-positive metastatic NSCLC. The drug inhibits ALK, insulin receptor, insulin growth factor receptor, and ROS1 (Khozin et al., 2015). In the ASCEND-5 trial, comparing ceritinib with chemotherapy in NSCLC, the former was associated with prolonged median PFS and better overall response (5.4 vs. 1.6 months; 39.1% vs. 6.9%, respectively) (Shaw et al., 2017). Furthermore, ceritinib showed improved outcomes in NSCLC as a first-line agent, compared to chemotherapy (Soria et al., 2017). In the first line setting, ceritinib was associated with higher frequency of diarrhoea, nausea, and vomiting, when compared to chemotherapy (85% vs. 11%; 69% vs. 55%; 66% vs. 36%, respectively). Furthermore, elevated enzymes related to hepatotoxicity were approximately three times more common in the ceritinib cohort (Soria et al., 2017). The role of cieritinib in a neoadjuvant setting still needs more investigation, but recent case report by Mai et al. (2022) demonstrated significant shrinkage of EML4-ALK positive adenosquamous carcinoma after two cycles of the TKI.

Ceritinib also shows important anti-cancer immunological effects. Adenosine signaling represents one of the mechanisms of immune escape performed by cancer cells. Schäkel et al. (2022) demonstrated that ceritinib inhibits ectonuclease CD39 in melanoma and triple negative breast cancer cells, which can contribute to the anti-cancer effects. In addition, in an *in vitro* experiment, Du and others showed that ceritinib decreases expression of PD-L1 in H2228 cells (NSCLC) through the inhibition of ERK (Du et al., 2020). Moreover, ceritinib was found to induce ICD in NPM1-ALK cells (Petrazzuolo et al., 2021) (Figure 2).

Other second-generation agents include alectinib, brigatinib, and ensartinib. Alectinib was approved by the FDA in 2015. The drug was developed for the treatment of patients with ALK receptor-positive NSCLC who are intolerant to crizotinib and whose disease has progressed (Larkins et al., 2016). Alectinib is a highly selective ALK inhibitor compared to crizotinib and is active against crizotinib resistance mutants (Sakamoto et al., 2011). The efficacy is related to its active metabolite M4, which suppresses cancer growth (Sato-Nakai et al., 2017). Inhibition of ALK autophosphorylation and phosphorylation of STAT3 and ALK (without ERK1/2) is the primary target of alectinib effective in NSCLC expressing EML4-ALK (Sakamoto et al., 2011). Alectinib penetrates the central nervous system (CNS), which is crucial for patients with brain metastases of NSCLC (Kodama et al., 2014; Ou et al., 2016). A recent retrospective study demonstrated superior real-world PFS in patients with advanced NSCLC treated with alectinib compared to crizotinib (Wang et al., 2023). Importantly, phase 3 trials (ALEX, J-ALEX, ALESIA) showed improved PFS in the alectinib cohort compared to crizotinib (Hida et al., 2017; Camidge et al., 2019; Zhou et al., 2019; Nakagawa et al., 2020). Moreover, the efficacy of chemotherapy versus alectinib in ALK-positive NSCLC patients previously treated with crizotinib was also compared. Alectinib has been proven to provide higher efficacy and longer PFS (Novello et al., 2018; Wolf et al., 2022). Additionally, alectinib may be used in a neoadjuvant setting. Loc et al. (2023) described 10 treatment-naïve patients treated with alectinib prior to surgery for advanced lung adenocarcinoma (stages III and IV). Fifty percent of patients showed a complete pathological response while major pathological response was detected in 90% of patients. As previously mentioned, in ALK positive patients treated with crizotinib, high expression of PD-L1 was associated with worse outcomes (Zhou et al., 2022). In contrast, PFS and OS of PD-L1 positive patients receiving alectinib did not significantly differ. Moreover, in the cohort of patients with PD-L1 tumor proportion score ≥50%, a positive trend regarding PFS was observed, but the result did not reach a statistical significance. ORR for PD-L1 negative and PD-L1 positive cohorts were 90% and 80.8%, respectively (Pan et al., 2023). As previously mentioned, alectinib shows a good penetration through blood-brain barrier. Thus, some studies evaluated its intracranial efficacy. For instance, Zou et al. (2022) described their experience with alectinib in patients with ALK-positive NSCLC and CNS metastases. These patients were divided into three cohorts, depending on their history of treatment. The first two groups involved patients, who were ALK-naïve or experienced an intracranial progression after treatment with crizotinib, respectively. The third cohort was composed of patients with CNS progression after treatment with other second generation ALK TKIs. In these groups, intracranial ORR was reported in 52.6%, 56.7%, and 33.3%, respectively (Zou et al., 2022).

Similarly to crizotinib, alectinib has also been evaluated in combination with agents targeting angiogenesis. Firstly, the protein expression of VEGF-A is elevated in NSCLC cells harboring ALK-rearrangement compared to control cell line. A preclinical study showed improved activity of combined alectinib with DC101, a VEGFR2 antibody in xenograft mouse models. Watanabe et al., demonstrated that above-mentioned combination was associated with a more significant tumor volume reduction in ALK-rearranged models, as compared to alectinib alone (Watanabe et al., 2021). In clinical settings, the combination of alectinib with bevacizumab has been investigated in an early phase trial. Eleven patients with ALK-rearranged advanced NSCLC have been evaluated. Importantly, 9 patients (81.8%) achieved an objective response and the median PFS was 19.8 months. Moreover, the ORR in 9 patients with CNS lesions was 77.8% (Lin et al., 2022). Another study investigated the use of alectinib with bevacizumab in 12 ALK-positive advanced (stages IIIB-IV) nonsquamous NSCLC patients who progressed on alectinib treatment. In contrast to the previous study, ORR was 8% and the median PFS was 3.1 months. However, a disease control rate was 67% and the median OS was 24.1 months. (Watanabe et al., 2022). These trials differed in case of efficacy. Nevertheless, the observed differences could result from baseline characteristics of included patients. In the former study, patients were ALK naive or received ALK TKIs other than alectinib. In contrast, the latter trial included patients previously treated with alectinib. Interestingly, a report by Nakasuka and others demonstrated that treatment with a combination of bevacizumab, pemetrexed and platinum promoted sensitivity to alectinib in primarily resistant patient with advanced ALK-positive NSCLC. However, the authors discovered that the tumor cells were alectinib sensitive prior to the initial treatment. Therefore, it was suggested that bevacizumab could stimulate the delivery of ALK TKI to the tumor (Nakasuka et al., 2019).

Ensartinib is another 2nd generation inhibitor which is not currently approved by the FDA. A phase 2 trial investigated the efficacy of ensartinib in a cohort of patients who progressed during the treatment with crizotinib. An objective response was observed in 52% of patients and the median PFS was 9.6 months. Importantly, an objective intracranial response and intracranial disease control were 70% and 98%, respectively (Yang et al., 2020). In addition, it showed an improved PFS compared to crizotinib as a first line ALK inhibitor in advanced, metastatic, or recurrent NSCLC cohort (Horn et al., 2021).

Brigatinib is an oral TKI approved by the FDA in 2020, which targets ALK and ROS1. Furthermore, *in vitro* studies showed a modest activity against FLT3, EGFR, insulin-like growth factor-1 receptor and INSR. Brigatinib inhibits ALK autophosphorylation and downstream phosphorylation of proteins such as AKT, STAT3, S6, and ERK1/2 (Hoy, 2021). Phase 3 ALTA-1L compared brigatinib to crizotinib in patients with advanced NSCLC, who did not previously receive an ALK inhibitor, and demonstrated a higher median PFS and ORR for brigatinib. Compared to crizotinib, brigatinib was associated with higher incidence (at least 5 percentage points) of increased blood creatinine kinase, cough, hypertension, and elevated lipase concentration. The percentage of patints who developed grade ≥3 AEs was slightly higher in the brigatinib group (61% vs. 55%), and the three most common serious AEs in this cohort included elevated blood creatinine kinase (16%), increased lipase level (13%), and hypertension (10%) (Camidge et al., 2018; Camidge et al., 2021). However, according to the ALTA-2 study, limited efficacy has been observed in case of NSCLC patients, who progressed on ceritinib and alectinib and were treated with brigatinib (ORR 26.2%, median PFS 3.8 months) (Ou et al., 2022). ALTA-3 trial compared the efficacy of brigatinib versus alectinib in NSCLC patients. The trial showed comparable efficacy between both TKIs (median PFS 19.3 months vs. 19.2 months for brigatinib and alectinib, respectively) (Chih-Hsin Yang et al., 2023). Furthermore, another study showed similar efficacy between both drugs in the first line setting in patients with ALK-positive advanced NSCLC in another study (Jeon et al., 2023). Second generation of ALK inhibitors are approved for the treatment of metastatic ALK positive NSCLC. Nonetheless, their efficacy has been detected in other diseases as well. For instance, brigatinib was effective in combination with chemotherapy and followed by stem cell transplantation in a pediatric patient with refractory ALCL (Caddeo et al., 2023).

### 3.3 Anaplastic lymphoma kinase–III generation inhibitor

Lorlatinib is a third generation ALK-TKI, which targets ALK and ROS1 and penetrates through blood-brain barrier. It was approved by the FDA in 2018 for patients with ALK-positive metastatic NSCLC, who progressed on first-line ALK inhibitors (crizotinib, alectinib, ceritinib) (Syed, 2019). Furthermore, recent study identified that lorlatinib induced tumor shrinkage in NSCLC patient with CLIP1-LTK fusion, indicating that it might also inhibit LTK kinase (Izumi et al., 2021). CROWN, a phase 3 clinical trial evaluating ALK-positive NSCLC patients, showed improved 12-month PFS and ORR with lorlatinib compared to crizotinib (80% vs. 35%; 76% vs. 58%, respectively). Furthermore, the study showed that the use of lorlatinib is significantly associated with hypercholesterolemia and hypertriglyceridemia, which respectively occurred in 70% and 64% of patients, as compared to 4% and 6% in the crizotinib cohort. The grade 3–4 severity of these AEs were 16% and 20%, respectively (Shaw et al., 2020). Recently, updated results from the CROWN study were published, which showed superior 3-year PFS in lorlatinib subgroup (64% vs. 19%) (Solomon et al., 2023). Moreover, the study also demonstrated significantly improved outcomes in patients with brain metastases (12-month PFS 78% vs. 22%) (Solomon et al., 2022). Different generations of ALK-TKIs can be used sequentially due to acquired resistance. Interestingly, patients who experienced resistance to lorlatinib may once again become sensitive for crizotinib and such therapy has been demonstrated in several reports (Shaw et al., 2016; Sakakibara-Konishi et al., 2019). Clinical efficacy of ALK-inhibitors in phase 3 trials has been summarized in Table 1.

**TABLE 1:** A list of selected phase 3 clinical trials evaluating the efficacy of ALK inhibitors in non-small cell lung cancer.

ALK-TKI	Name of the clinical trial/NCT registration number	ALK-naive	Arms	Number of patients	PFS (median months)	Response^a^
Crizotinib Shaw et al. (2013)	NCT00932893	Yes	Crizotinib	173	7.7	65%
			Chemotherapy	174	3	20%
Crizotinib Solomon et al. (2014)	PROFILE-1014	Yes	Crizotinib	172	10.9	74%
			Chemotherapy	171	7	45%
Ceritinib Shaw et al. (2017)	ASCEND-5	Patients after crizotinib	Ceritinib	115	5.4	39.1%
			Chemotherapy	116	1.6	6.9%
Ceritinib Soria et al. (2017)	ASCEND-4	Yes	Ceritinib	189	16.6	72.5%
			Chemotherapy	187	8.1	26.7%
Alectinib Camidge et al. (2019)	ALEX	Yes	Alectinib	152	34.8	82,9%
			Crizotinib	151	10.9	75,5%
Alectinib Hida et al. (2017); Nakagawa et al. (2020)	J-ALEX	Yes	Alectinib	103	34.1	92%
			Crizotinib	104	10.2	79%
Alectinib Wolf et al. (2022)	ALUR	Patients after crizotinib	Alectinib	79	10.9	50.6%
			Chemotherapy	40	1.4	2.5%
Ensartinib Horn et al. (2021)	NCT02767804	Yes	Ensartinib	143	25.8	74%
			Crizotinib	147	12.7	67%
Brigatinib Camidge et al. (2018); Camidge et al. (2021)	ALTA-1L	Yes	Brigatinib	137	24	71%
			Crizotinib	138	11.1	60%
Brigatinib Chih-Hsin Yang et al. (2023)	ALTA-3	Patients after crizotinib	Brigatinib	125	19.3	52%
			Alectinib	123	19.2	61%
Lorlatinib Shaw et al. (2020)	CROWN	Yes	Lorlatinib	149	-	76%
			Crizotinib	147	9.3	58%

^a^ the response includes an objective response rate or overall response, PFS, progression-free survival.

### 3.4 Novel anaplastic lymphoma kinase inhibitors

Iruplinalkib is a novel TKI, which suppresses ROS1 and ALK. The drug was found to inhibit phosphorylation of ALK, together with ERK and AKT (without STAT3) *in vitro* (NCI-H3122 cells). However, in an *in vivo* experiment (mouse NCI-H3122 xenograft model) iruplinalkib decreased phosphorylation of STAT3 and STAT5 (Yang et al., 2023). According to the phase 1 study by Shi et al., the use of iruplinalkib in ALK-rearranged patients resulted in objective response achieved in 58.2%. The ORR of 76% was observed in ALK-TKI naive patients. Among all patients, who received iruplinaklib, 97% of cases experienced AEs. The most common AEs (occurring in than 30% patients) were vomiting, nausea, hypercholesterolemia, and elevated hepatic enzymes (Shi et al., 2022). In a phase 2 study, advanced NSCLC patients with ALK-positive and crizotinib-resistant tumors were treated with iruplinalkib and ORR was 63% (Shi et al., 2023).

Lu and others developed XMU-MP-5, a new-generation ALK-TKI, which showed activity against EML4-ALK Ba/F3 cells. Moreover, in EML4-ALK Ba/F3, XMU-MP-5 inhibited ALK phosphorylation, together with downstream molecules (pSTAT3, pERK and pAKT). In addition, the novel drug inhibited proliferation of lung cancer cells (H3122), while reduced activity was observed in ALK negative lung cancer cells (A549, H1299, PC9) (L et al., 2022). Fang et al. described the development of APG-2449, which inhibits ALK, ROS1, and focal adhesion kinase (FAK). The authors observed that APG-2449 demonstrated antitumor properties in mice tumor models bearing different mutations (H3122 - EML4-ALK; KARPAS-299 - NPM-ALK; Ba/F3 - CD74-ROS1) (Fang et al., 2022).

Repotrectinib is another TKI, which targets ALK, ROS1, and TRK. In preclinical studies, it showed higher potency in EML4-ALK Ba/F3 cells, when compared to crizotinib. Furthermore, it presented similar activity to ceritinib, alectinib, and brigatinib, while it showed decreased potency compared to lorlatinib (Drilon et al., 2018). Moroever, repotrectinib showed antitumor efficacy in ALK-dependent neuroblastoma cells (Cervantes-Madrid et al., 2019). TPX-0131 and NVL-655 are two other drugs, which showed activity against wild type and mutant ALK, and are considered as a fourth generation ALK inhibitors (Murray et al., 2021; Pelish et al., 2021). Phase 1/2 clinical trial (NCT05384626) will evaluate dose-limiting toxicities, dosage, and ORR in ALK-positive advanced/metastatic NSCLC or other solid tumors (Inc, 2022). Furthermore, recent studies report several novel compounds/agents capable of inhibiting ALK (Wang et al., 2022; Cao et al., 2023; Fan et al., 2023).

## 4 Anaplastic lymphoma kinase inhibitors–resistance mechanisms

**FIGURE 3:**
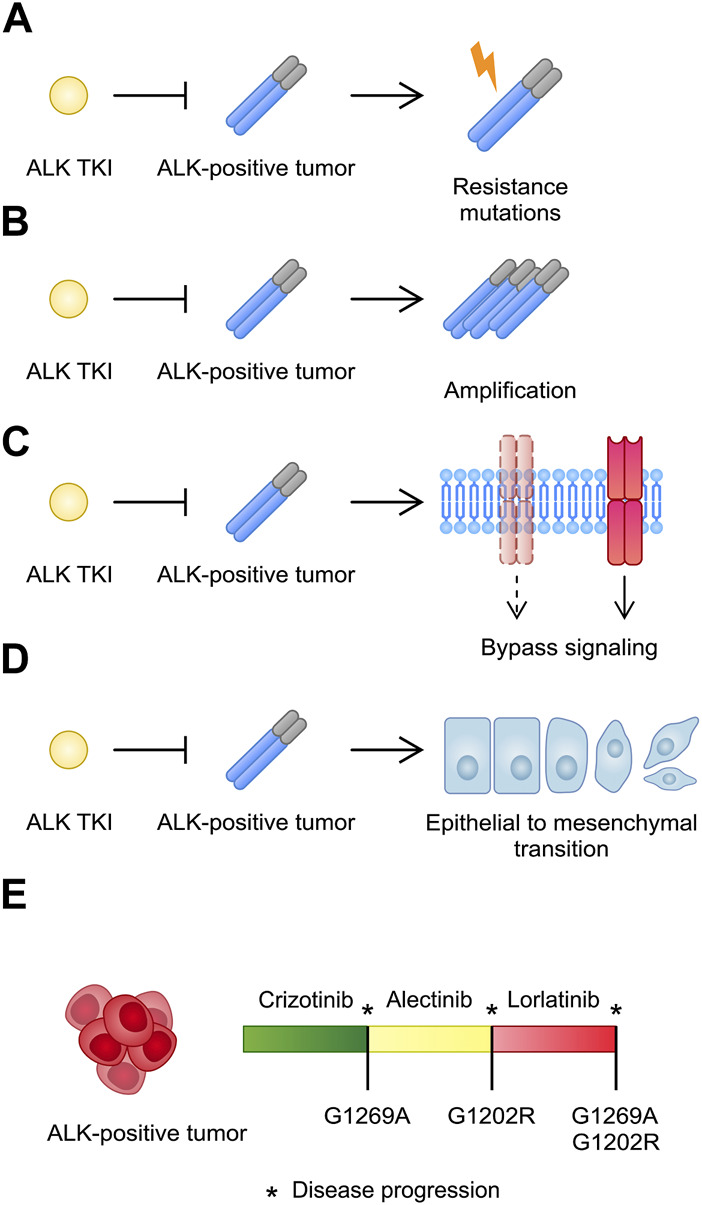
A schematic illustration of acquired resistance mechanisms to ALK inhibitors, including **(A)** resistance mutations; **(B)** amplification; **(C)** bypass signaling; **(D)** epithelial to mesenchymal transition. **(E)** A potential course of sequential treatment of ALK-positive cancer with a development of subsequent resistance mutations.

The use of ALK-TKIs is associated with acquired resistance, which may require the use of other treatment agent [median duration of response with crizotinib was 11.3 months according to the study by Solomon et al. (2014)]. Resistance may develop due to several mechanisms, such as secondary ALK mutation or amplification (Figure 3). Shaw et al. (2014) described 19 ALK-positive NSCLC patients with disease progression on crizotinib, who were later treated with ceritinib. In this subgroup, seven patients were identified with acquired ALK mutations or amplification. Interestingly, 12 patients did not have any additional genetic ALK aberrations. Katayama and others described 18 crizotinib-resistant ALK-positive NSCLC patients. High-level amplification was detected in only one patients while resistance mutations was identified in 4 patients (Katayama et al., 2012). These findings suggest that crizotinib might insufficiently inhibit ALK or that other mechanisms unrelated to ALK rearrangements might confer resistance. For instance, cancer cells might become stimulated through other tyrosine kinases, which is known as a bypass signal-induced resistance. In the case of ALK, activation of signaling pathways through EGFR, HER2, and HER3 with concomitant absence of ALK secondary mutations has been observed (Tanizaki et al., 2012). Tani et al. demonstrated that a knockdown of TGFα (a ligand of EGFR), restores the sensitivity to alectinib in resistant NSCLC cell line (H3122-AR) (Tani et al., 2016). EGFR signaling has been also found in lorlatinib-resistant cell lines (Katayama et al., 2023). Clinically, case reports showed detection of EGFR mutation in patients treated with various generations of ALK inhibitors. Michaux et al. demonstrated NSCLC patients, who were respectively sequentially treated with crizotinib + alectinib and alectinib + lorlatinib, and who developed EGFR mutations. Strategies used to overcome resistance included an introduction of brigatinib (shows activity against EGFR), a combination of ALK and EGFR inhibitors, and chemotherapy (Michaux et al., 2023). Thus, EGFR bypass is an important resistance mechanism which could develop after treatment with various generations of ALK-TKIs. Overcoming bypass resistance represents a significant clinical challenge. Introduction of dual inhibitors, which showed promising efficacy in *in vitro* experiments, could become a novel opportunity for these patients in the future (An et al., 2023). Interestingly, Talwelkar et al. demonstrated that combining ALK inhibitors with agents targeting its downstream molecule (PI3Kβ) is associated with an improved anticancer efficacy in ALK-rearranged NSCLC cell lines (Talwelkar et al., 2023). Similarly, a combination of crizotinib with duvelisib (PI3Kδ/γ inhibitor) increased anticancer efficacy in ALCL cell lines partially resistant to ALK inhibitors, as well as in an *in vivo* experiment (Mastini et al., 2023). In the case of enhanced HER3 phosphorylation as a resistance mechanism, a combination of pan-HER inhibitors, such as afatinib and dacomitinib, with lorlatinib might resensitize cells to the third generation ALK inhibitor, which has been recently demonstrated in an *in vitro* experiment (Taniguchi et al., 2023).

Additionally, an *in vitro* study showed that alectinib resistance is associated with increased phosphorylation of MET and elevated expression of hepatocyte growth factor (HGF). Inhibition of MET restored alectinib sensitivity. Moreover, a combination of metformin with alectinib lead to an interesting discovery of restoring alectinib sensitivity through the suppression of Gab1, an element of the HGF/MET signaling pathway (Chen et al., 2020). In clinical settings, Shiraishi and others have recently reported an ALK-positive NSCLC patient, who was treated with alectinib, lorlatinib, chemotherapy with carboplatin and nanoparticle albumin-bound paclitaxel, and bevacizumab, but the disease progressed. NGS analysis confirmed an amplification of the MET and HER2 genes. An introduction of crizotinib showed improvement for 4 months, until the disease progressed again (Shiraishi et al., 2023). Importantly, MAPK mutations also have been found to confer resistance to ALK inhibitor in neuroblastoma (Berko et al., 2023).

**FIGURE 4:**
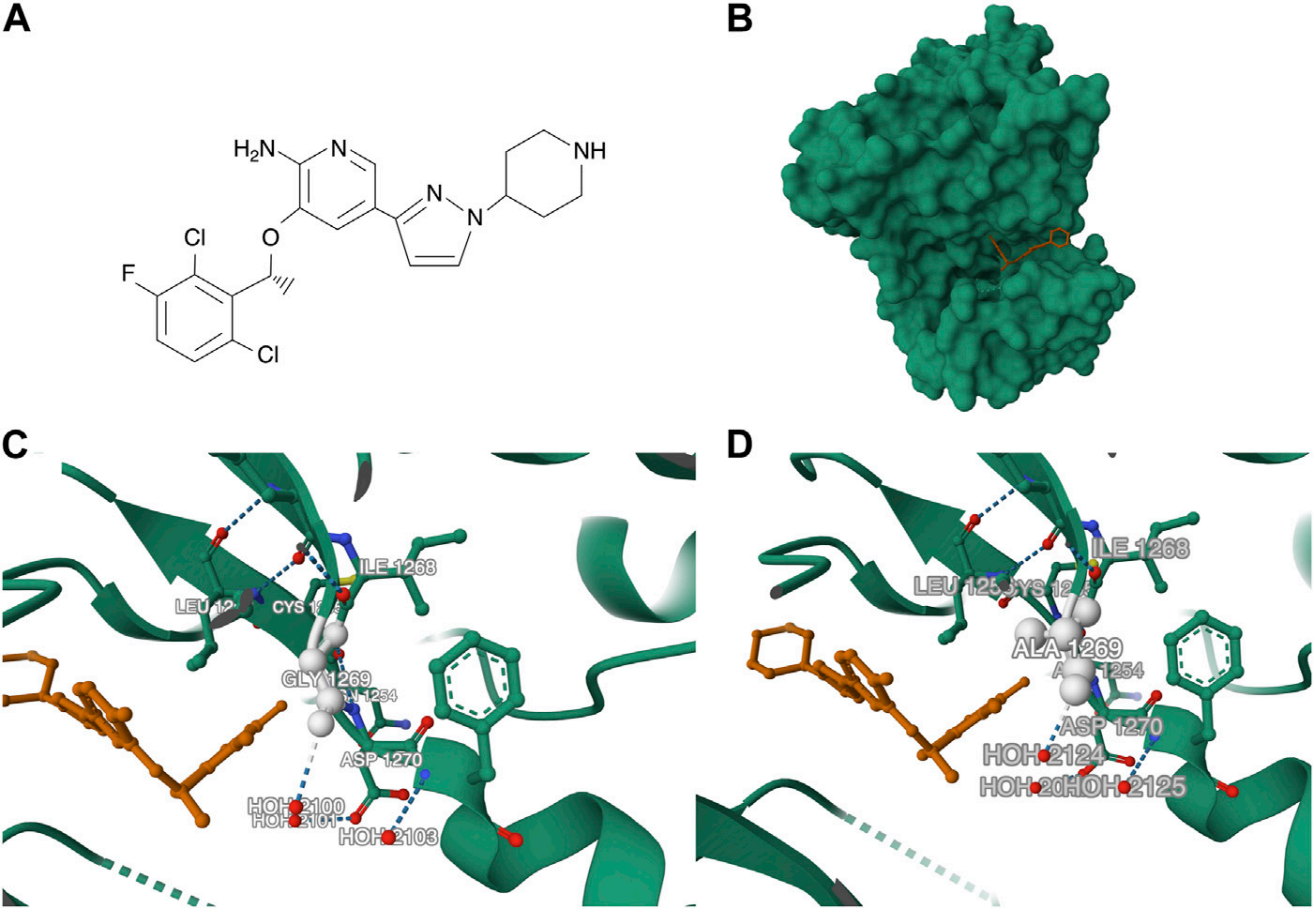
**(A)** Chemical structure of crizotinib. **(B)** Structure of the ALK in complex with crizotinib [PDB ID: 2XP2 (Berman et al., 2000; Cui et al., 2011; Sehnal et al., 2021)]. **(C)** Structure of the ALK in complex with crizotinib with focus on the G1269 region (PDB ID: 2XP2). **(D)** Structure of the G1269A mutant ALK [PDB ID: 4ANQ (Huang et al., 2014)].

Moreover, a study conducted by Gainor et al. (2016) investigated resistance mutations after treatment with various ALK-TKIs. L1196M and G1269A mutations were the most common among resistance mutations identified in crizotinib-treated patients (7% and 4%, respectively) (Figure 4). Other mutations included I1171T, E1210K, G1202R, S1206Y, and C1156Y. In addition, the authors evaluated mutation profiles after treatment with second-generation TKIs. After treatment with ceritinib (24 patients, 21 after previous crizotinib treatment), 54% and 17% presented single and several ALK resistance mutations, respectively. G1202R and F1174C/L were the most frequent (21% and 16.7%, respectively). Two cases with C1156Y mutations also harbored other ALK aberrations (I1171N; V1180L and G1202del). Among 17 patients treated with crizotinib and alectinib, 9 patients with ALK mutations were identified: G1202R (29%), I1171T/S (12%), V1180L (6%), and L1196M (6%). In the brigatinib cohort (5 out of 6 previously treated with crizotinib), G1202R mutation was the most common. Furthermore, the study showed that treatment with second generation of ALK TKIs is associated with more common ALK resistance mutations compared to crizotinib (Gainor et al., 2016). Shaw and others performed a similar study, additionally evaluating molecular analysis (tumor tissue, cell-free DNA) and its relationship with response to lorlatinib. The authors found that treatment with the third generation TKI was associated with ORR 73% of NSCLC patients previously treated with crizotinib. Response rate did not differ between cohorts with or without ALK mutation. In contrast, lorlatinib treatment in patients with ≥1 prior second generation ALK-TKI was correlated with better response in mutation-positive cohort (62% vs. 32%, respectively). Importantly, lorlatinib showed antitumor efficacy in the five most common resistance mutations (G1202R/del, F1174X, L1196M, G1269A, I1171X) (Shaw et al., 2019).

Therefore, acquired mutations are associated with various sensitivity profiles towards other ALK-TKIs. For instance, alectinib shows antitumor activity in cells with ALK G1269A mutation (Yoshimura et al., 2016). Moreover, ceritinib demonstrates efficacy against G1269A, I1171T, S1206Y, and L1196M mutations, among others (Friboulet et al., 2014). Importantly, novel ALK-TKIs demonstrate efficacy against resistance mutations as well. For instance, in ALK mutant Ba-F3 cell lines, XMU-MP-5 showed efficacy against G1269A and 1196M mutations, together with I1171T, C1156Y, and S1206Y. The half-maximal inhibitory concentration (IC_50_) of XMU-MP-5 in G1202R mutation was 49.4 nM. In contrast, in the same mutation, IC_50_ of crizotinib, alectinib and brigatinib was 206, 479 and 433 nM, respectively. XMU-MP-5 was inactive against cells harboring F1174L mutation (L et al., 2022). APG-2449 showed efficacy in mice with ALK L1196M mutation. Furthermore, it achieved better antitumor activity in G1202R mutations than crizotinib, ceritinib, ensartinib, and alectinib (Fang et al., 2022). Furthermore, in a study by Drilon et al., repotrectinib showed comparable activity to lorlatinib against EML4-ALK Ba/F3 cells harboring G1202R resistance mutation (Drilon et al., 2018). In another study, G1202R and I1171N mutants (alectinib-resistant) showed sensitivity to repotrectinib and ensartinib in Ba/F3 cells, respectively. The authors also predicted repotrectinib resistance mutations (G1202R + L1196M, F1174C/L/I, E1129V/K, C1156Y) (Doi et al., 2022).

Importantly, the majority of cells harboring single ALK mutations seem to be sensitive to lorlatinib (Gainor et al., 2016). However, L1256F represents a lorlatinib-resistant single mutation (Okada et al., 2019). Additionally, the development of resistance mutations seems dynamic, as they might become indetectable in plasma after introduction of higher generation TKI (Hua et al., 2022). Recent studies started to reveal multiple mutations, which drive resistance to the third generation TKI. For instance, G1269A + G1202R, I1171N + L1198F, and G1202R + S1206F + G1269A represent mutations identified in lorlatinib-treated patients. Importantly, Shiba-Ishii demonstrated that lorlatinib analogs might poses higher sensitivity towards these mutations (Shiba-Ishii et al., 2022). These mutations seem to develop more often in patients treated sequentially with first, second, and third lines of ALK-TKIs (Hua et al., 2022). Importantly, after occurrence of a compound mutation the patient may still respond to a previous-line ALK inhibitors. For instance, Takahashi et al. showed that Ba/F3 cells harboring I1171S + G1269A double mutation were resistant to crizotinib, alectinib and lorlatinib. In contrast, sensitivity was detected when cells were treated with brigatinib and ceritinib (Takahashi et al., 2020). Furthermore, lorlatinib-resistant patients may also benefit from re-administration of crizotinib. Sakakibara-Konishi et al. described a patient, who was treated with crizotinib, alectinib, and lorlatinib (as well as with standard chemotherapeutics), but subsequently progressed. In post-lorlatinib sample, the authors identified G1269A mutation, low ALK copy number gain, and MET amplification. Since crizotinib is a MET inhibitor, this alteration could have contributed to the resensitization (Sakakibara-Konishi et al., 2019). Moreover, Shaw et al. (2016) reported a patient who developed resistance to crizotinib (C1156Y mutation), but subsequently responded to lorlatinib. Nevertheless, resistance to the third generation TKI developed approximately 8 months later (L1198F mutation). Interestingly, the amount of tumor cells with C1156Y mutation substantially increased (50% after crizotinib treatment and nearly 100% after the development of lorlatinib resistance). A subsequent *in vitro* experiment revealed that Ba/F3 cells expressing ALK L1198F-C1156Y were resistant to second and third generation of ALK-TKI, but treatment with crizotinib showed good efficacy. Structural analysis revealed that L1198 is located close to ATP-binding site, which decreased lorlatinib binding affinity (Shaw et al., 2016). Interestingly, Mizuta and others demonstrated that some TKIs may demonstrate off target effects and present antitumor efficacy in cells with ALK resistance mutations. The authors found that gilteritinib, a TKI used to treat FLT3 (+) refractory acute myeloid leukemia, inhibited viabillity of Ba/F3 cells harboring EML4-ALK I1171N + F1174I mutations. Importantly, these cells were resistant to first, second, and third generation of ALK-TKI. Furthermore, gliteritinib inhibited I1171N + L1198H mutant, which partialy responded only to crizotinib and brigatinib. Moreover, among gliteritinib, alectinib and lorlatinib, low concentrations the former inhibited phosphorylation of ALK in I1171N + F1174I mutant overexpressed H3122 cells. Gliteritinib also showed good efficacy against number of single mutations resistant to second- or third-generation ALK inhibitors, such as I1171N or L1256F, as well as compound mutations resistant to lorlatinib (I1171N + L1256F) (Mizuta et al., 2021). Novel ALK TKIs represent a promising perspective regarding the compound mutations. For instance, Doi et al. showed that TPX-0131 inhibits mutations composed of G1202R with L1196M, E1129V, and C1156Y (Doi et al., 2022).

Nonetheless, ALK-rearranged tumors may still show resistance to all three generations of ALK-TKIs due to primary resistance mechanisms. For instance, simultanous mutations in other genes might confer such resistance. Kunimasa and others reported a patient with lung adenocarcinoma, who rapidly progressed on crizotinib (4 months), lorlatinib (1 month) and alectinib (1 month). NGS analysis revealed co-occurrence of PIK3CA E542K and RB1 L303V mutations in all sites (Kunimasa et al., 2021).

In addtion, resistance to ALK inhibitors may involve histological transformation. Fujita et al. reported a patient with metastatic lung adenocarcinoma, who was treated with crizotinib for 7 months, until the disease progressed. After standard chemotherapy, the patient was given alectinib. Surprisingly, metastatic lesions responded to treatment, but primary tumor progressed. Subsequent biopsy confirmed small cell lung cancer (Fujita et al., 2016). Transformation of metastatic lesion was recently reported by Xia and others (Xia et al., 2023). Importantly, both tumors remained ALK-positive after transformation. Recently, another report described a patient with small cell lung cancer transformation, which was followed by a NSCLC metastasis with I1171T mutation. To control both NSCLC and small cell lung cancer, the patient received temozolamide in combination with lorlatinib (Majeed et al., 2023). Moreover, a process of epithelial to mesenchymal transition (EMT) can play a role in ALK TKI resistance. EMT is an important mechanism taking part in embryogenesis. In tumorigenesis, EMT has been associated with the ivasiveness of cancer cells (Huang et al., 2022). Lung cancer resistant to crizotinib also showed a mesenchymal features. Importantly, silencing vimentin, a marker of a mesenchymal phenotype, improved the anti-cancer effects of ALK inhibitors (Kim et al., 2013). Furthermore, hypoxic condition could also contribute to the ALK TKI resistance through the induction of EMT (Kogita et al., 2014). Therefore, the disease can develop several resistance mechanisms to resist ALK inhibitors.

As demonstrated above, multiple studies have investigated resistance mechanisms in ALK-positive NSCLC. Nevertheless, the efficacy of ALK-TKI may differ in patients with less frequent ALK fusion partners. Kang eat al. described 11 NSCLC patients with huntingtin-interacting protein 1 (HIP1)-ALK rearrangements, among whom one patient with adenosquamous cell cancer harbored three fusions (EML4-ALK, HIP1-ALK, and ALK-PLEKHH20). Crizotinib was given to ten patients and in 9 cases an objective response was observed. Median OS and PFS were 58.8 and 17.9 months, respectively. First-line Lorlatinib was given to one patient, who achieved PR for over 26.5 months. Six variants of HIP1-ALK could be identified from these 11 patients: H21:A20, H28:A20, H30:A20, H19:A20, H19:A19, and H1:A16 (Kang et al., 2022). What’s more, it is unclear whether abundance of mutation contributes to the treatment sensitivity. In EGFR-mutated lung cancer, higher adjusted variant allele frequency (aVAF) has been associated with better efficacy of EGFR-TKI (Gieszer et al., 2021). According to Rao et al. (2023), aVAF was not correlated with PFS in first line crizotinib treatment. Furthermore, the authors did not find any correlations between PFS and ALK subclonality thresholds. Nevertheless, technical limitations of NGS might contribute to the negative results (Rao et al., 2023). In contrast, a recent study by Hizal et al. demonstrated that patients stratified by the expression of ALK-positive cells (NSCLC) respond differently to the first line alectinib treatment. Higher expression was associated with elevated ORR and PFS (Hizal et al., 2023). ALK VAF was found to correlate with clinical course in some patients with neuroblastoma. Berko and others showed that disease progression was associated with an elevation of VAF, while decrease or stable VAF level were observed in patients responding to lorlatinib. Nevertheless, a group of patients with small VAF and disease progression was observed as well, which might result from tumor heterogeneity or mutaitons of other genes (Berko et al., 2023). Furthermore, monitoring of interleukins may also help identify disease progression. Angeles and others investigated serum from ALK positive NSCLC patients treated with ALK inhibitors and found that elevation of IL-8, IL-6 and IL-10 were associated with disease progression (Angeles et al., 2023).

## 5 Conclusions and future perspectives

To conclude, targeted and individualized oncology represents a novel and hopeful trend in treating patients with neoplastic diseases. A recent study performed by Scott and others illustrates this sentence very well. The authors showed that inferior survival outcomes were achieved in NSCLC patients treated before the detection of actionable oncogenic drivers, and who were not subsequently switched to according TKIs, as compared to patients whose treatment began after the mutation report (Scott et al., 2023). To date, NSCLC remains the most studied ALK-positive cancer and good efficacy of ALK inhibitors resulted in approval of these agents for the treatment of metastatic NSCLC. Recently, crizotinib was also approved for the treatment of ALCL and IMT, which shows that ALK inhibitors can be beneficial in patients with various ALK-positive neoplastic diseases. However, it is worth mentioning that despite good and promising efficacy, these drugs may induce serious adverse reactions. Therefore, application of ALK inhibitors requires monitoring for drug-specific AEs. Furthermore, the treatment with ALK inhibitors is associated with acquired resistance. With the use of NGS and circulating tumor DNA (ctDNA), we can monitor genetic aberrations and detect resistance mutations. CtDNA molecules harbor genetic profiles of the primary tumor and can be identified in the blood. Therefore, monitoring ctDNA is an interesting and non-invasive method of molecular profiling (Cheng et al., 2016). For instance, ctDNA monitoring may be used to detect molecular residual disease after curative-intent first line treatment and to evaluate the risk of recurrence (Chaudhuri et al., 2017). Qiu et al. demonstrated that a positive ctDNA detection could precede the radiological recurrence of NSCLC by 88 days (Qiu et al., 2021). In patients treated with ALK inhibitors, the detection of ctDNA could identify a resistance mechanism and thus, discontinue the current treatment or apply another targeted agent (Guo et al., 2018).

Recent studies allowed us to understand the sensitivity profiles of these mutations to other treatment agents. Consequently, patients who progress on ALK inhibitors may still benefit from other mutation-sensitive agents. In addition, a detection of bypass signaling may require an introduction of other targeted therapies, which could prolong the response. Importantly, as sequential use of ALK inhibitors is correlated with occurrence of multiple mutations, further research should evaluate the sensitivity profiles of these genetic aberrations. Novel ALK-TKIs may be required to overcome the development of several mutations. Moreover, future studies should focus on investigating novel ALK-rearrangements and co-occurrence of mutations, which may drive a primary resistance to ALK inhibitors.
